# Modeling Health Event Impact on Smoking Cessation

**DOI:** 10.1155/2022/2923656

**Published:** 2022-02-27

**Authors:** Edwin D. Boudreaux, Erin O'Hea, Bo Wang, Eugene Quinn, Aaron L. Bergman, Beth C. Bock, Bruce M. Becker

**Affiliations:** ^1^Departments of Emergency Medicine, Psychiatry, And Quantitative Health Sciences, University of Massachusetts Medical School, Worcester MA, USA; ^2^Department of Psychology, Stonehill College, Easton MA, USA; ^3^Department of Quantitative Health Sciences, University of Massachusetts Medical School, Worcester MA, USA; ^4^Department of Mathematics, Stonehill College, Easton MA, USA; ^5^Departments of Emergency Medicine and Psychiatry, University of Massachusetts Medical School, Worcester MA, USA; ^6^The Miriam Hospital, Department of Psychiatry & Human Behavior, Warren Alpert School of Medicine, Brown University, Providence RI, USA; ^7^Behavioral and Social Science, The School of Public Health, Brown University, Providence RI, USA

## Abstract

**Background:**

This study examined how cognitive and affective constructs related to an acute health event predict smoking relapse following an acute cardiac health event.

**Methods:**

Participants were recruited from emergency departments and completed cognitive and emotional measures at enrollment and ecological momentary assessments (EMA) for 84 days postvisit.

**Results:**

Of 394 participants, only 35 (8.9%) remained abstinent 84 days postvisit. Time to relapse was positively associated with age, actual illness severity, self-efficacy, and quit intentions.

**Conclusions:**

Older, seriously ill patients with strong confidence and intentions to quit smoking remain abstinent longer after discharge, but most still relapse within three months.

## 1. Introduction

Despite the remarkable decreases in cigarette smoking rates since the 1960s, smoking remains one of the most significant preventable causes of death among Americans, accounting for around 480,000 deaths annually [1]. Unfortunately, the rate of smoking cessation has recently decreased, and smoking prevalence has stabilized at around 15.5% of the United States adult population, with no measurable changes since 2015 [2]. Moreover, remaining smokers disproportionally represent minority, uninsured, impoverished, and nonmetropolitan populations [3]. Continued research into understanding and promoting smoking cessation remains one of the most important public health priorities for the United States [4].

Studies have confirmed that both initial quit attempts and sustained smoking cessation occur at higher-than-expected rates after an individual experiences an acute health event, like myocardial infarction, or is diagnosed with a major medical illness, like cancer [5–12]. However, these studies also confirm that the positive effect of such a sentinel health event does not occur with every person, and, all too often, the change is short lived, with many smokers relapsing within only a few days or weeks after the event. Understanding the drivers of initial and long-term smoking cessation after an acute health event is important to inform interventions designed to maximize the “teachable moment” to get more smokers to attempt, to quit, and to help quitters transition to sustained abstinence. The sentinel event method [6] describes a systematic process for developing conceptual models to explain how health events can trigger positive behavior change. It is not a health behavior theory itself; rather, it provides a framework for identifying and testing cognitive, affective, and event-related constructs, typically by selecting and adapting good fit constructs from existing health behavior theories, and studying their associations with behavior change longitudinally.

Using the sentinel event method, O'Hea and colleagues [10] studied short-term smoking cessation over the 7 days after an ED visit for cardiac-related symptoms by selecting and testing constructs adapted from the theory of planned behavior [13] and self-regulation theory [14]. Their model emphasized the importance of behavioral intentions as a mediator between event-related constructs, such as perceived illness severity and smoking-related causal attributions, and smoking cessation, as well as teasing out the role of both cognitive and affective variables. Their results confirmed that intentions to quit smoking shortly before discharge were strongly associated with sustained abstinence over the 7 days after discharge and fully or partially mediated the associations between other predictors and abstinence. Furthermore, several novel findings were reported. *Perceived* illness severity, measured by assessing how serious the individual believed his/her illness to be, was positively associated with 7-day abstinence, even after controlling for *actual* illness severity, exhibiting a direct association with 7-day abstinence of nearly the same magnitude as quit intentions. However, the relationship was complex and depended heavily on the time anchor used for the perception ratings. Perceptions at initial symptom onset were more strongly associated with abstinence than perceptions upon initial arrival to the hospital or toward the end of the encounter. Moreover, the interaction between perceived illness severity and smoking-related causal attributions approached significance, suggesting that illness severity may have been more likely to inspire behavior change when the individual strongly believed the health problem was caused or aggravated by smoking. These findings, using more nuanced measurement of perceived illness severity over time than previous studies using aggregate measures [15, 16], may help explain the ambiguity in the literature surrounding this construct. Some studies have found it is important in predicting smoking cessation [10, 17], whereas others have not [18]. Severity perceptions are not static, and aggregating severity perceptions may hide or confound associations with intentions and behavior.

In sum, when a sentinel health event occurs, there is a complex relationship between actual illness severity, perceived severity, emotional reactions to the event, causal attributions, quit intentions, and subsequent smoking behavior. Further, the importance of perceived severity and illness-related emotions at different time anchors merits further evaluation, as these are not static constructs. The present study sought to expand the literature by examining constructs previously identified as a good fit for the population and research question [6, 10] using a sample of ED patients being evaluated for acute coronary syndrome. This study extended the predictor pool used by O'Hea and colleagues [10] by adding a measure of self-efficacy from social learning theory [19]. In this context, self-efficacy reflects the confidence in one's ability to quit smoking. Finally, the study design was improved by following the patients for a longer time period than reported by O'Hea and colleagues [10], extending the monitoring period to 12 weeks (84 days) rather than 7 days postdischarge. Smoking was tracked in a more precise way using ecological momentary assessment (EMA) six times/day for 28 days after the visit and a single end of day diary for the remaining 56 days, leading to daily tobacco use measures for 84 days. The feasibility of using EMA for tracking smoking in this opportunistic, heterogeneous sample was established in a pilot study, and the protocols were refined prior to study initiation [20]. Using EMA allowed for more precise measurement of time to lapse after discharge to create survival curves.

## 2. Method

### 2.1. Procedure

From September 2011 through March 2013, adult cigarette smokers with symptoms of acute coronary syndrome who presented to one of four hospitals between two health systems were considered for enrollment. To be eligible, patients had to be 18 years or older; have smoked at least 100 cigarettes; have chest pain, chest pressure, shortness of breath, or syncope as primary presenting symptoms; undergo a cardiac evaluation consisting of, at minimum, an electrocardiogram and cardiac enzyme test (troponin); and be an active smoker, defined as smoking 1 or more cigarettes per day. Patients were ineligible if they presented with illicit drug use or alcohol abuse, had chest pain resulting from trauma, or were unable to be interviewed (e.g., severe medical illness, cognitive insufficiency, or insurmountable language barrier).

Participants were recruited during their ED or cardiac inpatient stay as close to the end of their medical visit as possible. This ensured a majority of the acute health event had already occurred prior to the interview, which was consistent with study interest in how perceptions and emotions changed over the course of the event. The range in actual illness severity in the patients who enrolled in the study was extensive, with some patients being discharged home from the ED with no evidence of cardiac disease (less serious) and others being diagnosed with an acute myocardial infarction requiring multivessel cardiac arterial bypass graft (CABG) surgery (very serious).

In addition to the baseline interview, participants were given a study cellphone and were expected to complete up to six random, interactive voice response (IVR) EMA calls per day, with each survey lasting one to two minutes, for 28 days, followed by a single end of day diary call for 56 additional days totaling 84 days of monitoring. Participants were trained on using the cellphone for EMA calls before discharge. A research assistant completed a structured follow-up telephone interview at 28 days, three months, and six months, though for the purposes of this paper, the focus will be entirely on the EMA data for the 3-month window.

Participants could keep the cellphone (or $40 if using their own cellphone) and had a free nationwide cell service for the duration of the study. They were compensated $0.25 per completed call for the EMA and $1.00 per completed end of day diary call in the last 56 days, for a total of up to $98.00. Withdrawn participants returned the cellphone but were paid for completed surveys. Additionally, participants received $15.00 compensation for completing research assistant telephone interviews, up to $45.00. The study was approved by the Institutional Review Boards of the hospitals, and all patients signed informed consent.

### 2.2. Measures

At the time of enrollment, participants completed measures developed to assess a range of cognitive and affective constructs as described below. The psychometric properties of these scales are published by Boudreaux and colleagues [21], and they are the same used by O'Hea and colleagues [10]. For two of these constructs, perceived illness severity and illness-related emotions, participants were asked to provide a rating for three specific time anchors: time anchor 1, when they first started experiencing symptoms; time anchor 2, when they first arrived at the hospital; and time anchor 3, currently, at the time of enrollment. The purpose of measuring items at each time anchor was to assess whether perceived illness severity and illness-related emotions fluctuated over the course of the healthcare encounter as hypothesized in previous papers [6, 17, 18]. For the entire health event to be chronicled, participants were enrolled as close to discharge as possible. Therefore, the third anchor rating, “right now,” typically occurred after most of the health event was over and within 24 hours of the patient's discharge. The first two anchors, by necessity, were retrospectively rated, the limitations of which are described under limitations.

#### 2.2.1. Actual Illness Severity

Enrolled participants' medical diagnoses were highly heterogeneous in severity. A categorical disease severity rating, consistent with common clinical distinctions, was assigned to each participant. These categorical ratings were coded as follows: (0) treated in the ED and discharged, (1) admitted to an inpatient floor but received no intervention, and (2) admitted with an intervention, including percutaneous stent, balloon angioplasty, or CABG.

#### 2.2.2. Perceived Illness Severity

Perceived illness severity was measured using three different time anchors, describe above. Participants' responses to four questions that assessed perceived illness severity were rated on a five-point Likert-type scale: 1 = “strongly disagree,” 2 = “disagree,” 3 = “neither agree or disagree,” 4 = “agree,” and 5 = “strongly agree.” Sample items include “Something is seriously wrong with me,” and “I have a life-threatening illness.”

#### 2.2.3. Illness-Related Negative Emotions

Illness-related negative emotions were measured using three different time anchors. Participants' responses to eight items were rated on a five-point Likert-type scale: 1 = “not at all,” 2 = “a little,” 3 = “moderately,” 4 = “quite a bit,” and 5 = “extremely.” The emotions rated were anxiety, fear, frustration, nervousness, sadness, hopelessness, stress, and anger.

#### 2.2.4. Smoking-Related Causal Attribution

Participants' beliefs about the association between smoking and their current health problem were measured using four items rated on a five-point Likert-type scale: 1 = “strongly disagree,” 2 = “disagree,” 3 = “neither agree or disagree,” 4 = “agree,” and 5 = “strongly agree.” Sample items include “My current illness is due to a health problem caused by smoking,” and “Quitting smoking could improve my health.”

#### 2.2.5. Self-Efficacy

Self-efficacy was assessed using a 0- to 10-point confidence ruler assessing confidence in quitting smoking within the next month, with 0 = not at all confident and 10 = 100% confident [22].

#### 2.2.6. Nicotine Dependence

Participants' nicotine addiction severity was measured using the Heaviness of Smoking Index [23, 24].

#### 2.2.7. Intention to Quit

Using two different time anchors (time anchor 2 and 3), participants rated five questions that assessed intention to quit smoking using a five-point Likert-type scale: 1 = “strongly disagree,” 2 = “disagree,” 3 = “neither agree or disagree,” 4 = “agree,” and 5 = “strongly agree.” Example items include “I intend to quit smoking sometime within the next 30 days,” and “I have decided to quit smoking today.”

#### 2.2.8. Tobacco Cessation Outcomes

Tobacco use was measured by EMA. At each assessment, participants rated whether or not he/she had smoked that day, and, if so, provided an estimate of the number of cigarettes smoked since awakening. The tobacco outcome data was transformed to hours to relapse, defined as the hours from discharge from the ED or inpatient unit to the time when the participant smoked his/her first cigarette. This variable was then transformed to form two categorical variables: (1) a dichotomous variable denoting sustained abstinence for the first 7 days after discharge (yes or no), which allowed comparison with extant literature; and (2) a 6-category ordinal variable based on the timing of the relapse: 1 (within 24 hours), 2 (between 1 and 7 days), 3 (between 8 and 14 days), 4 (between 15 and 28 days), 5 (between 29 and 56 days), and 6 (did not lapse during the 84 day window, i.e., sustained abstinence throughout the 3-month monitoring period).

#### 2.2.9. Other Variables

We measured age, sex, race, ethnicity, and state of residence.

### 2.3. Data Analyses

Data analyses proceeded in several stages. First, the association between all of the bivariate predictors and 7-day abstinence status was calculated. Second, Pearson (for continuous variables) or Spearman (for ordinal variables) correlation analyses were conducted to examine the associations among the predictors, interactions (multiplicative) between causal attribution and perceived severity and illness-related negative emotions, and time to lapse expressed as the 6-point ordinal variable. Correlation analysis was performed using SAS 9.4 statistical software package [25].

**Figure 1:**
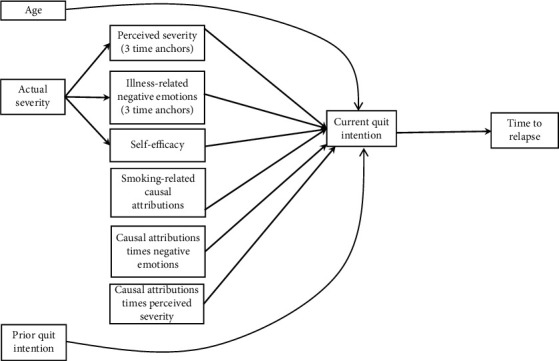
Hypothesized initial model of the relationships between the constructs of theory of planned behavior and quit intention and time to relapse.

Third, a path analysis was conducted to test the hypothesized model presented in Figure 1 that specifies relationships between all observed variables, interaction variables, and the 6-point ordinal outcome, using Mplus [26].

Mediation effects were tested using the Sobel test, a commonly used method for testing the significance of the mediation effect [27]. Standardized regression coefficients for all paths were estimated using maximum likelihood (ML) estimation. Missing data was handled using full information maximum likelihood (FIML). Goodness of model fit was assessed using absolute and comparative fit indices, including chi-square to degrees-of-freedom ratio (*χ*^2^/df), root mean square error of approximation (RMSEA), Bentler's comparative fit index (CFI), and the Tucker Lewis index (TLI) [28]. Acceptable model fit is determined by an RMSEA less than 0.08 and values of CFI and TLI greater than 0.90 [29].

## 3. Results

### 3.1. Descriptives

**Figure 2:**
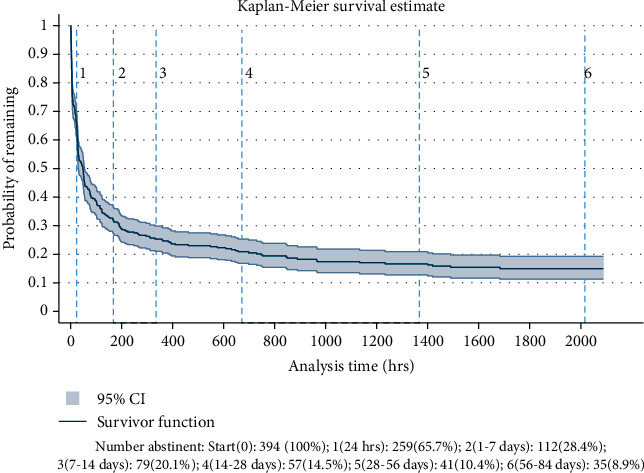
Kaplan-Meier survival curve of the entire sample.

**Figure 3:**
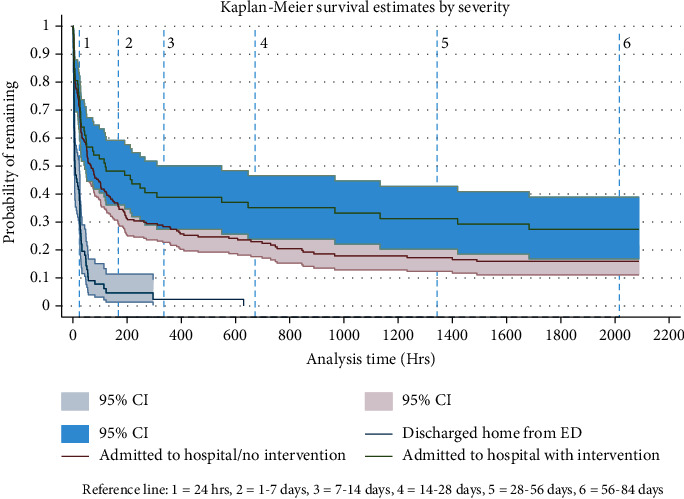
Kaplan-Meier survival curve of subsamples based on actual illness severity.

Of the 434 patients enrolled, 40 withdrew from the study or provided insufficient EMA compliance to determine the date and time of first lapse, leaving 394 participants for analysis (see Table 1). Participants' ages ranged from 18 to 84 years old, with an average age of 53, and 200 (51%) were male. Most (354; 91%) were white, 35 (9.0%) were black, and 31 (8.0%) identified as Hispanic/Latino. Median compliance with the EMA was 69.9% (IQR: 46.1% and 85.3%). Figures 2 and 3 plot survival curves for the entire sample and actual severity subgroups.

Of those who lapsed, average time to lapse was 146.6 hours (SD = 283.0 hours), or roughly 6 days. Sustained abstinence 7 days postdischarge was achieved by 112/394 (28.4%). Using the 6-category lapse variable, 135 (34.3%) lapsed within 24 hours of discharge, 147 (37.3%) lapsed between one and seven days, 33 (8.4%) lapsed between seven and 14 days, 22 (5.6%) lapsed within 15 and 28 days, 16 (4.1%) lapsed between 29 and 56 days, 6 (1.5%) lapsed between 56 and 84 days, and 35 (8.9%) remained abstinent the entire 84 days.

### 3.2. Predicting 7-Day Abstinence

Table 1 shows the bivariate relations between the predictors and initial 7-day sustained abstinence. The strongest predictors were as follows: actual severity (*χ*^2^ = 32.94, *p* < 0.001), self-efficacy (*t* = 8.34, *p* < 0.001), current intentions to quit (*t* = 7.77, *p* < 0.001), state of site (*χ*^2^ = 7.70, *p* = 0.006), smoking-related causal attribution (*t* = 4.45, *p* < 0.001), and age (*t* = 3.52, *p* < 0.001).

### 3.3. Bivariate Correlations with Time to Relapse

The bivariate associations between the predictors, quit intention, and time to relapse expressed as the 6-category ordinal variable were examined using Pearson or Spearman correlation coefficients (see Table 2). Time to relapse was most strongly correlated with current quit intention (*r* = 0.34, *p* < 0.001), self-efficacy (*r* = 0.34, *p* < 0.001), actual severity (*r* = 0.30, *p* < 0.001), the interaction between smoking-related causal attribution and perceived severity (*r* = 0.20, *p* < 0.001), age (*r* = 0.20, *p* < 0.001), smoking-related causal attribution (*r* = 0.18, *p* < 0.001), highest value of perceived illness severity (*r* = 0.11, *p* < 0.05), and negative emotions at illness onset (*r* = −0.11, *p* < 0.05). Current quit intention was associated with the same variables as the time to relapse, with the addition of prior quit intentions (*r* = 0.51, *p* < 0.001), the interaction between causal attribution and illness-related negative emotions (*r* = 0.25, *p* < 0.001), average perceived severity (*r* = 0.21, *p* < 0.001), perceived severity at hospital presentation (*r* = 0.18, *p* < 0.001), and perceived severity at discharge (*r* = 0.10, *p* < 0.05).

### 3.4. Path Analyses of the Hypothesized Relationships

The initial hypothesized model is displayed in Figure 1. We hypothesized that actual severity would have a direct effect on perceived illness severity, illness-related negative emotions, and self-efficacy, which in turn would have an effect on current quit intention and time to relapse. Age, smoking-related causal attribution, and prior quit intention were hypothesized to have direct effect on current quit intention, which in turn would have an effect on time to relapse. The interactions of causal attribution × perceived severity and causal attribution × negative emotions were hypothesized to have a direct effect on current quit intention. Estimation of this initial model revealed an unacceptable model fit which suggested the need for modification of the initial model.

**Figure 4:**
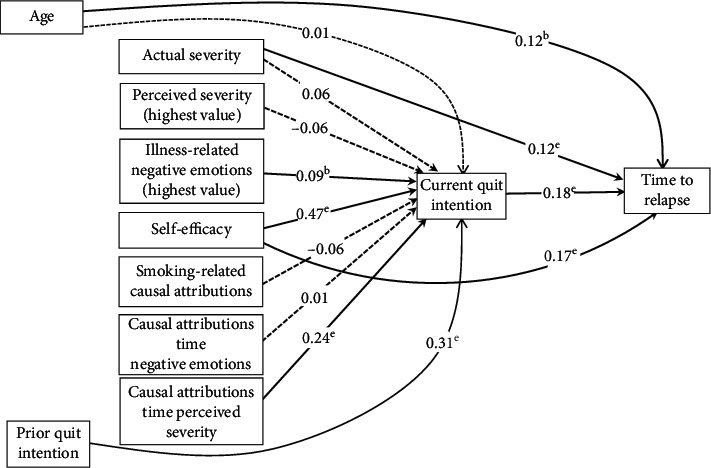
Revised structural model showing direct effects of actual and perceived severity, negative emotions, self-efficacy, casual attributions, and prior quit intention on current quit intention, which in turn has direct effect on time to lapse. Standardized path coefficients are shown. The highest values of perceived severity and negative emotions across times 1, 2, and 3 were used. *p* < 0.05, *p* < 0.01, and *p* < 0.001.

In modifying the initial path model, we included perceived illness severity, illness-related negative emotions, and self-efficacy as exogeneous variables and allowed these variables to covary with actual severity. Rather than all three time anchors for perceived illness severity and illness-related negative emotions, we used each individual's highest rating from the three time anchors for each construct. The overall fit of the revised model (Figure 4) was excellent.

The chi-square/df ratio was 1.93, the RMSEA was 0.05, the SRMR was 0.03, the CFI was 0.99, and the TLI was 0.99. Illness-related negative emotions, self-efficacy, the interaction between causal attributions and perceived severity, and prior quit intention predicted a high level of current quit intention, which in turn predicted longer time to relapse. In addition, age, self-efficacy, and actual severity had a direct effect on time to relapse. The analysis revealed an *R*^2^ value of 0.57 for current quit intention and of 0.20 for time to relapse.

The Sobel test of mediation effects indicated that current quit intention mediated the relationship between self-efficacy and time to relapse (*z* = 2.96, *p* = 0.003) and the relationship between prior quit intention and time to relapse (*z* = 2.88, *p* = 0.004). In addition, the Sobel test suggested that current quit intention mediated the relationship of the interaction between smoking-related causal attributions and perceived severity with time to relapse (*z* = 2.78, *p* = 0.005).

### 3.5. Perceived Illness Severity and Illness-Related Negative Emotions at Times 1, 2, and 3

**Figure 5:**
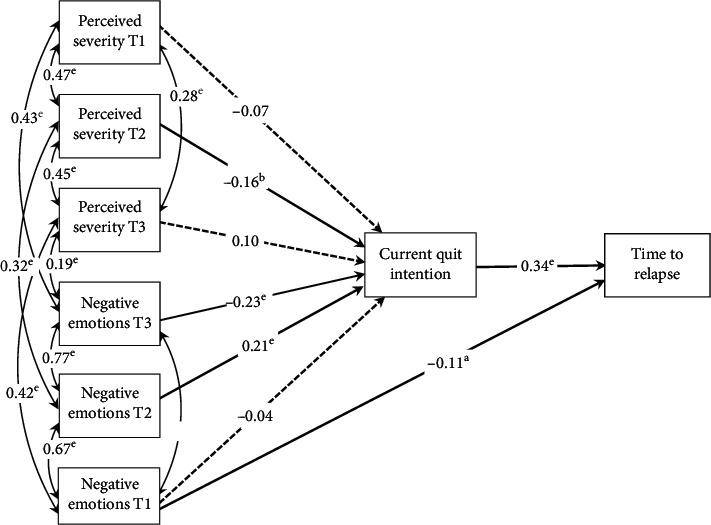
Revised structural model showing direct effects of perceived severity and negative emotions at times 1, 2, and 3 on current quit intention, which in turn has direct effect on time to lapse. Standardized path coefficients are shown. Solid lines represent statistically significant relations; dotted lines represent nonstatistically significant relations. *p* < 0.05, *p* < 0.01, and *p* < 0.001.

Because measuring constructs across different time anchors is an innovative and emerging research area, a reduced model including only perceived severity and emotions at times 1, 2, and 3, current quit intention, and time to relapse was constructed (Figure 5).

The overall fit of this model was excellent. The chi-square/df ratio was 1.31 (*p* = 0.256), the RMSEA was 0.03, the SRMR was 0.02, the CFI was 0.98, and the TLI was 0.95. Perceived severity and illness-related negative emotions at time 2 predicted a high level of current quit intention, which in turn predicted longer time to relapse. Illness-related negative emotions at time 3 were negatively associated with current quit intention, and illness-related negative emotions at time 1 have a direct negative effect on time to relapse. The analysis revealed an *R*^2^ value of 0.07 for current quit intention and of 0.13 for time to relapse, respectively.

Our structural equation model includes 9 manifest variables and two interaction terms. Based in the rule/recommendations of *n* = 10–20 observations per variable [29, 30], our study with a sample size of 394 achieves adequate statistical power to detect true relationships in the data. This is also evidenced by our excellent model fit.

## 4. Discussion

Our study is the most comprehensive evaluation of the cognitive and affective factors associated with an acute cardiac health event and their relationship with smoking one week and three months after the event. Our findings confirmed many previously identified patterns and extended our understanding by evaluating new associations. Consistent with the literature, most of our sample of acute cardiac patients initiated smoking cessation upon discharge, but the vast majority relapsed back to smoking relatively soon after the visit. Specifically, 66% went at least 24 hours immediately after discharge without smoking, but only 28% went 7 days without smoking, and around 9% remained continuously abstinent for 84 days. It is challenging to directly compare these rates with rates among smokers in the general community, because comparable methods of identifying a given day when a person is not having a health event and following them for a similar period have not been used; however, large epidemiological studies suggest that only 3–5% of community-based smokers typically initiate smoking cessation in a given year [31–33], which is markedly less than those in our sample with a quit attempt. Our data confirm that an acute healthcare encounter can be a sentinel event that inspires changes in smoking, but it also confirms that relapsing back to smoking remained the pronounced norm.

Importantly, while relapsing back to smoking was the norm, this did not occur equally across our sample; instead, there were important predictors of short-term and longer abstinence. Greater actual illness severity was one of the most powerful predictors of abstinence, as shown in Figure 3. Those suffering a myocardial infarction who were admitted and received a cardiac intervention, like a percutaneous stent or CABG, were more likely to be abstinent than those who were less ill. Approximately 44% of such seriously ill patients achieved 7-day abstinence and 30% remained abstinent at 84 days. In contrast, those discharged home from the ED, i.e., the less severe group, revealed a very steep relapse curve, with only 4% achieving 7-day abstinence and 100% back to smoking by 28 days. Those who were admitted for observation but received no intervention fared somewhere in between. This pattern replicates the literature on illness severity and smoking cessation [34, 35].

Quit intentions prior to discharge and self-efficacy both exhibited strong bivariate associations with 7-day abstinence (Table 1) and time to relapse (Table 2). In the final structural equation model (Figure 4), quit intentions continued to have significant direct associations with time to relapse, and self-efficacy had both a direct effect on time to relapse as well as a mediated effect through increased intentions to quit. These findings are consistent with the corpus of literature relating these two constructs to smoking-related behavior change [36–39] and reinforces their fundamental importance for understanding both short-term and medium-term abstinence after an acute health event. Despite their preeminence in theory-based research and their conceptual incorporation into motivational interventions [40], empirical support for whether such interventions actually lead to increased intentions and self-efficacy and whether these changes, in turn, drive greater likelihood of abstinence are surprisingly rare [41]. Such mechanisms of action studies are crucial to testing whether quit intentions and self-efficacy can be influenced by intervention and are causal drivers behind improved abstinence.

Other illness-related variables demonstrated complex patterns with abstinence. Smoking-related causal attributions showed strong bivariate associations with intentions to quit, 7-day abstinence, and time to relapse. Generally speaking, those individuals who believed their illness was due to their smoking reported stronger intentions to quit and were more likely to be abstinent at 7 days and had longer time to relapse. Moreover, the interaction between causal attribution and the highest perceived illness severity was strongly associated with quit intentions and time to relapse at the bivariate level. In the final structural equation model, the main effect of smoking-related causal attribution no longer predicted quit intentions or time to relapse but the interaction term remained a predictor of quit intentions. Put differently, after all variables were entered into the model, it was not simply whether the individual thought his/her illness was related to smoking that drove quit intentions; instead, those that perceived they had a very serious illness and who thought this serious illness was due to smoking were most likely to intend to quit. This was similar to findings reported by O'Hea and colleagues [10] who found the interaction between smoking-related causal attribution and perceived severity at illness onset predicted 7-day abstinence after discharge in a similar sample of cardiac patients. Our results generally support other studies that have established a positive association between perceived severity and subsequent smoking cessation [10, 17, 42, 43]. However, some studies have not found this association [18, 44]. Future studies should continue to pay close attention to perceived illness severity, including assessing these perceptions at different time anchors and evaluating the interaction between smoking-related causal attributions and perceived severity.

Illness-related negative emotional reactions, or what colloquially might be considered a “health scare,” were expected to promote abstinence. While previous studies have suggested negative affect may instigate relapses back to smoking among quitters [45], other studies of acute health events have suggested illness-related negative emotions might instigate increased motivation to quit, which in turn leads to increase probability of smoking cessation [10, 17, 18]. However, the associations in our data were complex and, in some cases, contradictory. At the bivariate level, illness-related negative emotions did not demonstrate strong associations with 7-day abstinence or with time to lapse, with the exception of one *negative* association between initial illness-related negative emotions and time to lapse, which was the opposite of what was expected. The more detailed model examining ratings from each time anchor (Figure 5) demonstrates further complexity. Illness-related negative emotions at illness onset were *negatively* associated with time to relapse, and, similarly, illness-related negative emotions at discharge were *negatively* associated with current quit intentions, while, in contrast, negative emotions at hospital arrival were *positively* associated with quit intentions. When all variables were entered into the final full model, the highest value of illness-related negative emotions modestly predicted higher quit intentions. It is difficult to reconcile these seemingly contradictory associations with the extant literature, because most studies have not included measures of emotions related to the health event. O'Hea and colleagues [10] generally found that illness-related negative emotions were associated with stronger quit intentions, with illness-related negative emotions at hospital presentation also being associated with greater abstinence over the 7-day postdischarge period. Further study is required to fully understand the strength and direction of the association between illness-related negative emotions, quit intentions, and abstinence.

There were several limitations associated with this study. It is impossible to collect ratings of perceived illness severity, emotions, or quit intentions at the time when symptoms first began since these invariably occur hours to days *prior to* presenting to the emergency department. It is likewise not possible to collect ratings in real time immediately upon presentation, because the patient must be triaged and stabilized before research staff approach the patient. As a result, we must invariably rely on patient recall for these initial time anchors. This limitation is partially mitigated by the fact we approached patients during their healthcare encounter, minimizing the recall bias that might occur if we had obtained ratings days or weeks after they were discharged.

Patients who are admitted to an inpatient unit are forced to quit smoking. As a result, these participants would have already had up to several days of abstinence prior to discharge. This forced abstinence is not the same as voluntary cessation, so we framed the intentions to quit smoking within the context of their discharge home. It is possible, however, that this period of abstinence influenced their ratings. In addition, pinpointing exactly when the individual had their first cigarette was important, but compliance with EMA was variable. Some individuals were removed from the analysis because of lack of compliance, which may have reduced the sample's representativeness. In addition, for some individuals, hours or days may have transpired between the actual lapse and their recording of the lapse. Because of the frequency of the assessments, it was unlikely this was longer than 24 hours. While it would be preferable to have precise measures immediately after the lapse, our data remains much more precise than most traditional studies that evaluate abstinence using time period separated by weeks or months. Finally, we did not collect biochemical verification of abstinence, because it was not feasible to do so. This is partially mitigated by the fact that the pressure to exaggerate abstinence is low in noninterventional studies like this one.

## 5. Conclusion

Our results confirmed the pattern of high lapse back to smoking after an acute health event and the preeminence of several key constructs, including actual illness severity, intentions to quit smoking, and smoking-related self-efficacy, in predicting abstinence for the 7 days after discharge, as well as time to lapse over the 84 days after discharge. Our results also revealed complex associations between illness-related cognitive factors and emotions, quit intentions, and tobacco use outcomes. The patterns reinforced the importance of taking measures of these variables at different time anchors and exploring their interactions.

## Figures and Tables

**Table 1:** Individual characteristics of study sample (*n* = 394).

Variables	Overall	Relapsed within 7 days	Did not relapse in 7 days	*t* or *χ*^2^	*p*
Sample size (*n*)	394	282 (71.5%)	112 (28.4%)		
Age	53.3 (10.8)	52.2 (10.8)	56.3 (10.3)	3.52	<0.001
Sex					
Male	200	151 (75.5%)	49 (24.5%)	3.08	0.079
Female	194	131 (67.5.4%)	63 (32.5%)		
Race					
White	354	250 (70.6%)	104 (29.4%)	1.07	0.587
Black	35	27 (77.1%)	8 (22.9%)		
American Indian/Alaska native	1	1 (100.0%)	0 (0%)		
Hispanic/Latino	31	27 (9.6%)	4 (3.6%)	3.985	0.060
Site					
Rhode Island	173	111 (64.2%)	62 (35.8%)	7.70	0.006
Massachusetts	217	167 (77.0%)	50 (23.0%)		
Actual severity					
Discharged home from ED (low)	77	74 (96.1%)	3 (3.9%)	32.94	<0.001
Admitted but no intervention (medium)	245	168 (68.6%)	77 (31.4%)		
Admitted with intervention (high)	72	40 (55.6%)	32 (44.4%)		
Perceived severity					
Perceived severity at time 1	3.5 (1.1)	3.5 (1.1)	3.4 (1.2)	0.83	0.407
Perceived severity at time 2	3.9 (0.9)	3.8 (0.9)	4.0 (0.9)	2.19	0.029
Perceived severity at time 3	3.4 (1.0)	3.3 (1.0)	3.6 (1.0)	2.18	0.030
Highest value of perceived severity	4.1 (0.8)	4.1 (0.8)	4.3 (0.7)	2.22	0.030
Average value of perceived severity	3.6 (0.8)	3.5 (0.8)	3.7 (0.8)	1.37	0.171
Event related negative emotions					
Negative emotions at time 1	2.8 (1.1)	2.8 (1.1)	2.7 (1.1)	1.04	0.298
Negative emotions at time 2	2.8 (1.1)	2.8 (1.1)	2.8 (1.1)	0.02	0.981
Negative emotions at time 3	2.2 (1.1)	2.2 (1.1)	2.2 (1.2)	0.13	0.900
Highest value of negative emotions	3.1 (1.1)	3.1 (1.0)	3.1 (1.1)	0.02	0.987
Average value of negative emotions	2.6 (1.0)	2.6 (1.0)	2.6 (1.0)	0.44	0.657
Causal attributions of smoking to current illness/health status	3.9 (1.0)	3.7 (1.0)	4.2 (0.8)	4.45	<0.001
Causal attributions × highest score of perceived severity	16.1 (5.3)	15.3 (5.2)	18.0 (5.1)	4.70	<0.001
Causal attributions × highest score of negative emotions	12.0 (5.4)	11.5 (5.1)	13.1 (5.9)	2.49	0.014
Heaviness of smoking index (HSI)	2.7 (1.4)	2.8 (1.3)	2.6 (1.5)	1.03	0.305
Self-efficacy	6.1 (3.0)	5.5 (2.9)	7.8 (2.3)	8.34	<0.001
Prior intentions to quit smoking	3.0 (1.1)	2.9 (1.0)	3.0 (1.2)	0.611	0.271
Current quit intention	3.6 (1.1)	3.4 (1.0)	4.2 (0.8)	7.77	<0.001

**Table 2:** Correlation coefficients between perceived severity, negative emotions, causal attributions, social norms, heavy smoking index, intentions to quit smoking, and time to relapse (*n* = 394).

Variables	1	2	3	4	5	6	7	8	9	10	11	12	13	14	15	16	17	18	19	20
1. Current quit intention	1.00																			
2. Time to relapse^∗^	0.34^c^	1.00																		
3. Age	0.12^a^	0.20^c^	1.00																	
4. Perceived severity at time 1	0.02	-0.07	-0.03	1.00																
5. Perceived severity at time 2	0.18^c^	0.10^a^	0.11^a^	0.47^c^	1.00															
6. Perceived severity at time 3	0.10^a^	0.09	0.06	0.28^c^	0.45^c^	1.00														
7. Average perceived severity	0.12^a^	0.05	0.05	0.78^c^	0.80^c^	0.74^c^	1.00													
8. Highest value of perceived severity	0.21^c^	0.11^a^	0.10^a^	0.55^c^	0.84^c^	0.61^c^	0.85^c^	1.00												
9. Negative emotions at time 1	0.01	-0.11^a^	-0.10^a^	0.42^c^	0.25^c^	0.19^c^	0.38^c^	0.26^c^	1.00											
10. Negative emotions at time 2	0.08	-0.02	-0.08	0.25^c^	0.32^c^	0.22^c^	0.34^c^	0.30^c^	0.77^c^	1.00										
11. Negative emotions at time 3	-0.05	-0.03	-0.09	0.20^c^	0.21^c^	0.42^c^	0.36^c^	0.22^c^	0.60^c^	0.67^c^	1.00									
12. Average negative emotions	0.02	-0.06	-0.10^a^	0.33^c^	0.30^c^	0.31^c^	0.40^c^	0.29^c^	0.89^c^	0.92^c^	0.86^c^	1.00								
13. Highest value of negative emotions	0.04	-0.02	-0.08	0.29^c^	0.33^c^	0.24^c^	0.37^c^	0.32^c^	0.87^c^	0.91^c^	0.69^c^	0.93^c^	1.00							
14. Self-efficacy	0.67^c^	0.34^c^	0.06	0.01	0.14^b^	0.09	0.10^a^	0.17^c^	0.01	0.09	-0.01	0.03	0.08	1.00						
15. Causal attributions of smoking to current illness/health status	0.39^c^	0.18^c^	0.22^c^	0.02	0.12^a^	0.14^b^	0.12^a^	0.15^b^	0.14^b^	0.13^b^	0.01	0.10^a^	0.15^b^	0.27^c^	1.00					
16. Actual severity	0.21^c^	0.30^c^	0.25^c^	-0.03	0.15^b^	0.19^c^	0.13^b^	0.20^c^	-0.11^a^	-0.07	-0.12^a^	-0.11^a^	-0.07	0.21^c^	0.22^c^	1.00				
17. Heaviness of smoking index (HSI)	0.01	-0.05	0.05	-0.11^a^	-0.07	0.02	-0.07	-0.01	-0.01	-0.01	-0.03	-0.02	0.01	-0.06	0.18^c^	0.07	1.00			
18. Prior intentions to quit smoking	0.51^c^	0.01	0.02	0.10^a^	0.08	0.07	0.11^a^	0.06	0.11^a^	0.13^b^	0.09^a^	0.13^b^	0.11^a^	0.42^c^	0.14^b^	-0.07	-0.12^a^	1.00		
19. Causal attributions × highest score of perceived severity#	0.41^c^	0.20^c^	0.22^c^	0.31^c^	0.54^c^	0.42^c^	0.54^c^	0.64^c^	0.25^c^	0.27^c^	0.13^b^	0.24^c^	0.29^c^	0.30^c^	0.84^c^	0.27^c^	0.13^b^	0.14^c^	1.00	
20. Causal attributions × highest score of negative emotions#	0.25^c^	0.09	0.07	0.22^c^	0.31^c^	0.24^c^	0.33^c^	0.32^c^	0.72^c^	0.75^c^	0.51^c^	0.74^c^	0.82^c^	0.21^c^	0.65^c^	0.07	0.11^a^	0.16^c^	0.67^c^	1.00

*p* < 0.05. *p* < 0.01. *p* < 0.001. ^∗^Time to lapse expressed as 6 point ordinal variable.

## Data Availability

Upon request submitted to the corresponding author (edwin.boudreaux@umassmed.edu), the complete deidentified raw data, syntax file, and log files for analysis will be shared.
